# Mixtum: a graphical tool for two-way admixture analysis in population genetics based on *f*-statistics

**DOI:** 10.1093/bioinformatics/btag123

**Published:** 2026-03-13

**Authors:** José-María Castelo, José-Angel Oteo, Gonzalo Oteo-García

**Affiliations:** Instituto de Ciencia de los Materiales (ICMUV), Universitat de València, Paterna 46980, Spain; Departament de Física Teòrica, Universitat de València, Burjassot 46100, Spain; Institute for Integrative Systems Biology I2SYSBIO, Paterna 46980, Spain; Dipartimento di Biologia Ambientale, Sapienza Università di Roma, Rome 00185, Italy; Centre for Palaeogenetics, Stockholm University, Stockholm SE-106 91, Sweden; Department of Archaeology and Classical Studies, Stockholm University, Stockholm SE-106 91, Sweden

## Abstract

**Summary:**

*Mixtum* is a Python-based code that estimates ancestry contributions in a process of two-way admixture based on bi-allelic genotype data. The outcomes of *Mixtum* come from the geometric interpretation of the *f*-statistics formalism. Designed with user-friendliness as a priority, *Mixtum* allows to interactively handle a menu of user-supplied populations to build different mixture models in conjunction with the set of auxiliary populations required by the framework. The results are presented graphically and numerically. Importantly, *Mixtum* provides a novel index (an angle) that assesses the quality of the ancestral reconstruction of the model under scrutiny. The use and interpretation of the outcomes of *Mixtum* are explained and illustrated with case studies.

**Availability and implementation:**

The open source code is available on GitHub at https://github.com/jmcastelo/mixtum and on Zenodo at https://doi.org/10.5281/zenodo.17789375. *Mixtum* is implemented in Python and runs on Linux, Windows and macOS.

## 1 Introduction

Given a triplet of populations characterized by Single Nucleotide Polymorphisms (SNP), *Mixtum* determines whether two of them can be the parental populations, or ancestry sources, of the third, which represents the result of an admixture process. We aim to provide this tool as a user-friendly method to evaluate admixture scenarios where the main mathematical difficulty stems from the fact that the sampled proxies are genetically distant from the ancestral triplet, as they have accumulated the effect of genetic drift. The so-called *f*-statistics formalism (Patterson *et al.* 2012, Haak *et al.* 2015, Lazaridis *et al.* 2016, Peter 2016, Lipson 2020, Harney *et al.* 2021, Oteo-García and Oteo 2021, Peter 2022, Oteo and Oteo-García 2024) is a method that reduces the evolutionary genetic drift contributions and provides estimates of the mixing coefficient.

*Mixtum* implements part of a geometric interpretation of the methodological framework originally introduced in Oteo-García and Oteo (2021) and Oteo and Oteo-García (2024). What follows is a brief overview of its mathematical foundation. For additional details and mathematical reasoning behind the method, readers should refer to the original publications.

Each population is interpreted as a point in a space in which the axes represent the allele frequencies of the SNPs genotypes. The dimension of this frequency space is the number of SNPs in the dataset. At the beginning of an admixture process the three points are aligned, with the admixed population in between, closer to the main donor in case of asymmetric contributions. The relative distances are in correspondence with the admixture proportion α. After that, genetic drift causes these three points to follow Brownian-like trajectories over time and to acquire a triangular disposition, which is the situation expected with the proxy data. The key of *f*-statistics stays in the approximate restoration of the ancestral alignment, removing the effect of genetic drift in the three populations. To this end, a number of auxiliary populations are required in the dataset, which are also referred to as outgroups or reference populations in the literature.

Any misalignment in the allele frequency space can be quantified by the angle at the admixed population vertex. This is done in two scenarios. We can measure: (i) an angle ϕ (termed pre-JL) directly with the proxies, and (ii) an angle φ (termed post-JL) using the approximate ancestral alignment provided by *f*-statistics. This way, the closer φ is to $180^{{}^{\circ}}$, the better the ancestral alignment reconstruction; and the closer ϕ is to $180^{{}^{\circ}}$, the smaller the effect of genetic drift. The difference φ−ϕ>0 accounts for the amount of genetic drift removed. Conversely, φ<ϕ indicates an incorrect admixture model. The post-JL angle φ is a novel contribution to the field of *f*-statistics (Oteo and Oteo-García 2024).

**Figure 1:**
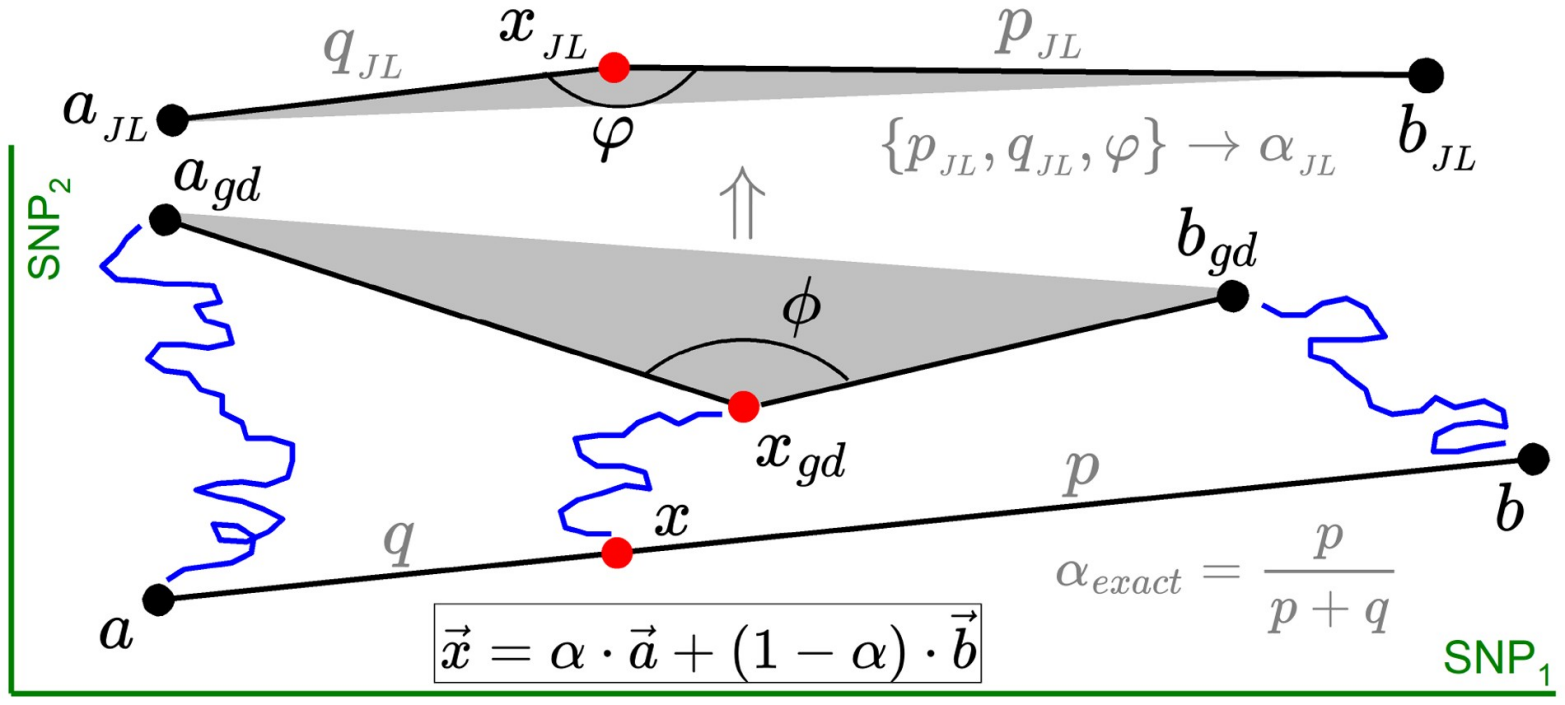
2D allele frequency space. The collinear shape *axb* of the admixture triplet (admixture beginning) becomes the triangle $a_{gd}x_{gd}b_{gd}$ (proxies) as a consequence of genetic drift (crinkly trajectories). Top: shape after JL projection.

Figure 1 provides a schematic representation of these ideas in an allele frequency space with two SNPs: the initial admixture alignment *axb* (*a* and *b* sources) becomes the triangle shape $a_{gd}x_{gd}b_{gd}$ as the result of genetic drift (blue lines). At the top, the shape of an approximate ancestral alignment $a_{JL}x_{JL}b_{JL}$ provided by *f*-statistics after reducing the effect of genetic drift. The estimation of α with $a_{gd}x_{gd}b_{gd}$ (pre-JL) is much worse than with $a_{JL}x_{JL}b_{JL}$ (post-JL).

## 2 Materials and methods

*Mixtum* has been written in Python 3, depends on *pyside6* for its GUI elements, on *numpy* as numerical library and on *matplotlib* for plotting. The code is released under the GPLv3 license. Both a script for command-line execution and a visual GUI version are provided. Computations may be run using multiple parallel processes, leveraging modern CPU capabilities, and the results can be exported as data files. To replicate a computation done on the GUI, one can export the arguments required to run the analogous command-line script, along with the populations used.

*Mixtum* expects to read a dataset in EIGENSTRAT format (Patterson *et al.* 2012), which is composed of a triad of plain text files. One contains the genotypes for the individuals in the dataset. The second contains information identifying individuals and the population they belong to. The third contains the SNPs identification. PACKEDANCESTRYMAP format is also supported.

A poorly sequenced population may significantly decrease the effective number of SNPs in the allele frequency table, since every time a missing SNP value appears in all individuals of a particular population, that SNP has to be discarded for all the selected populations.

### 2.1 What Mixtum computes

The formulas that give rise to *Mixtum* results can be expressed in terms of the *f*-statistics $f_{2},f_{3}.f_{4}$, plus the renormalized $f_{4}^{\prime }(a,b;i,j)=\sqrt{s}f_{4}(a,b;i,j)/\sqrt{f_{2}(i,j)}$; where *s* stands for the effective number of SNPs in the allele frequency table.

#### 2.1.1 The estimate of α

An admixed population *x* is represented by the population vector ${\vec{p}}_{x}$ and is considered as a linear combination of two sources, ${\vec{p}}_{a}$ and ${\vec{p}}_{b}$: ${\vec{p}}_{x}=\alpha {\vec{p}}_{a}+(1-\alpha ){\vec{p}}_{b}$, where α∈[0,1] is the contributing proportion of donor *a*. This vector expression stands for a linear system of *s* equations with one unknown, α, and must be solved using least squares or a similar method. In *f*-statistics, the system is first projected into a random subspace [referred to as dimensional reduction into the Johnson-Lindenstrauss (JL) subspace], whose axes are generated by the difference vectors of pairs of *m* auxiliary populations, *i* and *j*, and then solved using least squares. The method can be implemented by calculating the slope α of the linear fit to the set of points $\{f_{4}^{\prime }(a,b;i,j),$  $f_{4}^{\prime }(x,b;i,j)\}$:


(1) $$\alpha =\frac{\sum_{i<j}^{m}f_{4}^{\prime }(x,b;i,j)f_{4}^{\prime }(a,b;i,j)}{\sum_{i<j}^{m}f_{4}^{\prime 2}(a,b;i,j)},\;\alpha \in [0,1],$$

which is denoted $\alpha_{JL}$ in Fig. 1.

#### 2.1.2 The estimate of angles ϕ and φ

The pre-JL angle ϕ is given by


(2) $$\text{cos}\;\phi =\frac{f_{3}(a,b;x)}{{\left[f_{2}(a,x)f_{2}(b,x)\right]}^{1/2}}.$$

The value $\phi =180^{{}^{\circ}}$ marks the start of the mixing process. The admixture scenario holds while $\phi >90^{{}^{\circ}}$. This constraint implies $f_{3}(a,b;x)<0$, the conventional admixture test.

The post-JL angle reads


(3) $$\text{cos}\;\varphi =\frac{\sum_{i<j}^{m}f_{4}^{\prime }(x,a;i,j)f_{4}^{\prime }(x,b;i,j)}{{\left[\sum_{i<j}^{m}f_{4}^{\prime 2}(x,a;i,j)\sum_{i<j}^{m}f_{4}^{\prime 2}(x,b;i,j)\right]}^{1/2}}.$$

ϕ<φ indicates the successful reduction of the effect of genetic drift after JL projection. Furthermore, the closer the selected donors are to the exact ones, the closer φ will be to $180^{{}^{\circ}}$. An admixture model is ruled out if φ<ϕ (see the third case study).

### 2.2 The set of auxiliary populations

The most relevant pairs of auxiliary populations, with regard to determining α, are those with large $\left|f_{4}(a,b;i,j)\right|$ as they have more weight in determining the slope in (1) (see the first case study). Below is a heuristic tip for interactively refining the set of auxiliary populations using *Mixtum* in relation to the presence of outliers in the regression plot.

### 2.3 The number of SNPs

**Figure 2:**
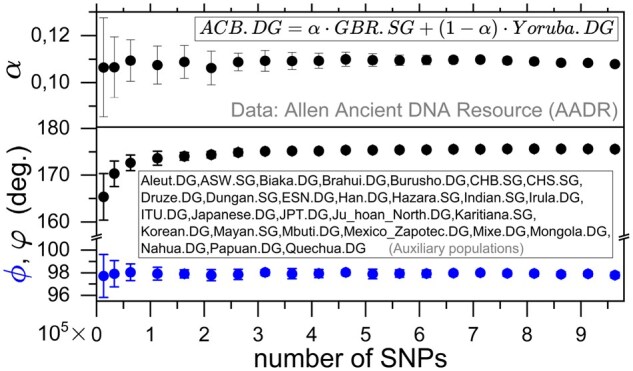
Dependence on the number of SNPs of the post-JL α and φ (top), and the pre-JL ϕ (bottom).

The JL dimensional reduction involved in *f*-statistics relies on the high dimension of the allele frequency space. For the second case study below, Fig. 2 shows the way α, ϕ and φ depend on the number of SNPs, i.e. the dimension of the allele frequency space. Each point is the average of fixed-size blocks of adjacent SNPs, whose origins are randomly located in the SNP sequence. The error bar is one standard deviation.

## 3 How to Mixtum

In the *Input files* tab the triad of data files is loaded and then parsed and checked for a valid structure. A populations file can be loaded and modified later on in the *Populations* tab. The number of SNPs to be processed can be limited. The allele frequencies table is then built up and can be saved in plain text format with the populations as columns and their labels in the header. The number of processors may be adjusted to compute in parallel. In the *Admixture model* tab, the model is set. The remaining populations may be selected at will to act as auxiliary, with a lower bound equal to four; or eight, if bootstrap is enabled. All the numerical outcomes can be exported. The task is then to interpret the results:

1. **Regression plot tab**. (i) The slope (1) (color bar) has to be α∈[0,1], for a model to be valid. Consider the two error bars: the first from the dispersion of points in the linear fit and the second from the bootstrap on auxiliary populations. (ii) Outliers in the plot: removing certain auxiliary populations (click on the points to identify them) refines the determination of α and φ.
2. **Angles tab**. (i) The closer φ is to $180^{{}^{\circ}}$, the better the reconstruction of the ancestral alignment. The error bar comes from bootstrapping auxiliary populations. (ii) ϕ<φ indicates reduction of the effect of genetic drift. (iii) The conventional admixture test is $\phi >90^{{}^{\circ}}$ (which implies $f_{3}(a,b;x)<0$), but there may be cases $\phi <90^{{}^{\circ}}$ which, after JL projection, appear as admixture scenarios, i.e. $90^{{}^{\circ}}<\varphi$. (iv) A model is ruled out whenever ϕ>φ.
3.
$f_{4}$ -**ratio histogram tab**. Obtained from (1) with m=2, the $f_{4}$-ratio is a well-known statistic related to the estimation of α. This distribution is usually quite dispersed. Some auxiliary populations may give values out-of-range. This tab is intended to illustrate the potential misinterpretation from using single values of the $f_{4}$-ratio, not as a tool to measure α.
4. **PCA tab**. The set of populations to be projected can be selected from those in the allele frequency table. An admixture triplet tends to appear aligned in a PCA projection (Peter 2022). It can be also used to gain insight about the auxiliary populations that generate outliers in the fit.
5. *f*-**statistics tab**. Specific values of $f_{2}$, $f_{3}$ and $f_{4}$, can be computed.
6. **Output logs**. Main results log includes: (i) admixture model, number of SNPs and auxiliary populations, (ii) α given with 95% CI error bars obtained from the linear fit (the first) and from the bootstrap on auxiliary populations (the second). (iii) ϕ without error estimate and φ with 95% CI error bar from auxiliary populations bootstrap, and (iv) below, tidbits: the pre-JL admixture proportion, a statistical average of (in range) $f_{4}$-ratios and the $f_{3}$ admixture test.

## 4 Case studies

**Figure 3:**
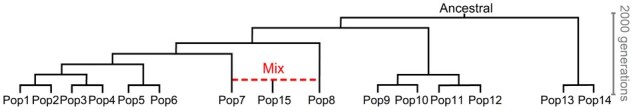
Simulated phylogenetic graph (*msprime*).

**Figure 4:**
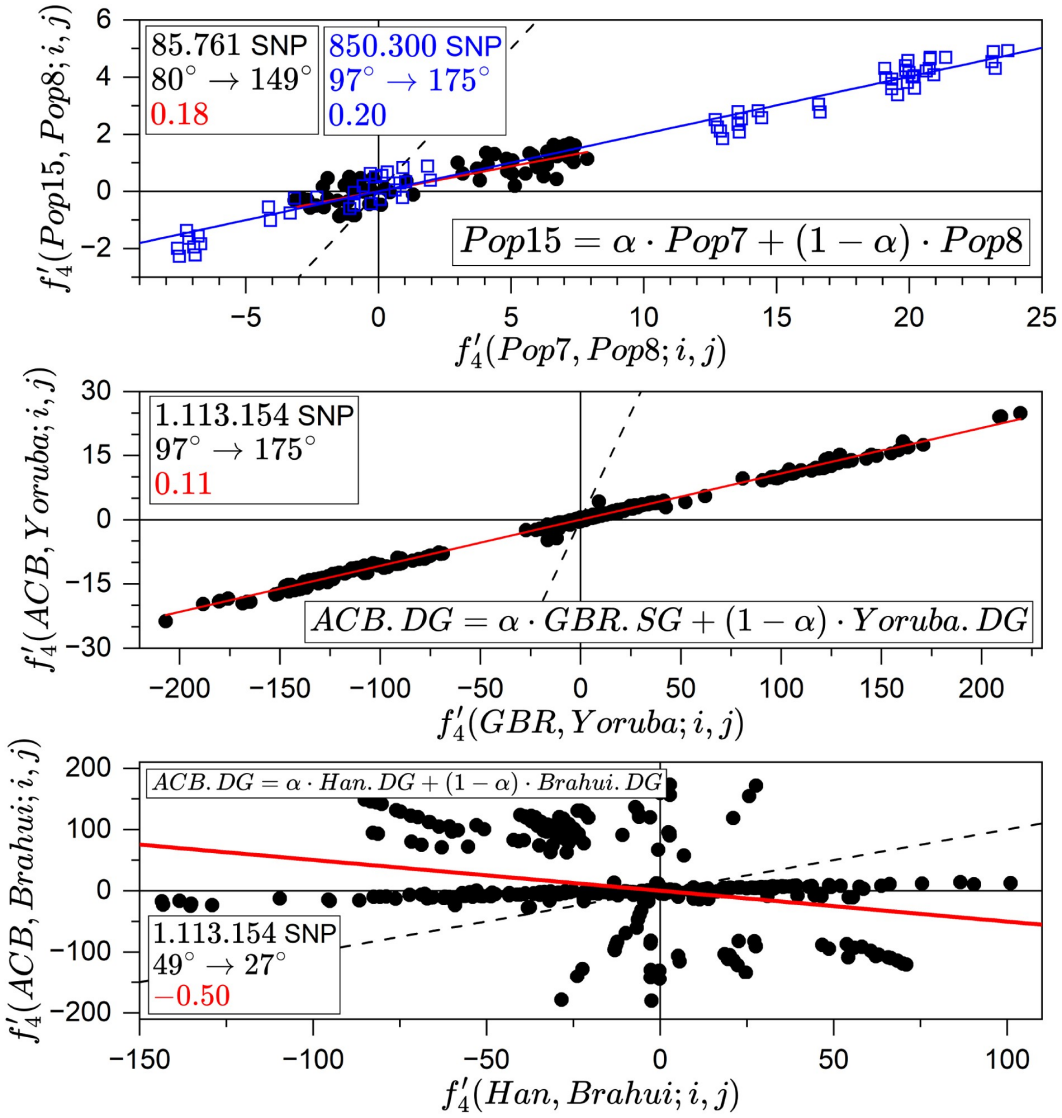
Linear fits for case studies. The legends contain information about the admixture model, the number of SNPs, the pre-JL and post-JL angles, and the slope of the fit (solid lines). Auxiliary populations i,j, are those in Fig. 2. The dashed line has a slope equal to one.

Three case studies are presented below. The first is based on the simulated phylogeny in Fig. 3 (data available with the code) generated with *msprime* (Baumdicker *et al.* 2022). The next two are cases with real data from the Allen Ancient DNA Resource (AADR) V9 (Mallick *et al*. 2024), with 1 113 154 SNPs. The outcomes are gathered in Table 1 as well as in the panels of Fig. 4.

**Table 1:** Two-way admixture models.^a^

Mix	Donor 1	Donor 2	Angle (degrees)		$\alpha \pm \Delta_{1}\pm \Delta_{2}$
			ϕ	$\varphi \pm \Delta_{2}$	
Pop15	**Pop7**	**Pop8**	80	149±18	0.18±0.03±0.05
	Pop6	Pop8	77	132±35	0.20±0.04±0.10
	Pop1	Pop8	76	118±42	0.27±0.09±0.15
	Pop7	Pop10	66	69±97	0.16±0.20±0.30
	Pop7	Pop14	68	66±84	0.20±0.24±0.36
	Pop14	Pop8	71	39±30	−0.30±0.19±0.69
	Pop7*	Pop8*	81	170±5	0.20±0.01±0.02
ACB	GBR.SG	Yoruba.DG	97	175±3	0.108±0.001±0.007
	Han.DG	Brahui.DG	49	27±18	−0.5±0.2±0.8

a Top: simulated data. Nominal models are boldfaced. Number of SNPs is 85 761, except 850 300 in the last line (*). Bottom: Real data from with 1 113 154 SNPs.

*1. msprime* simulated phylogeny

The dataset has 15 populations with 10 individuals each and 85 761 SNPs. We study the model Pop15=α·Pop7+(1−α)·Pop8, which has nominal value α=0.2. In addition, we have studied the variants: Pop15=α·Pop7+(1−α)·Any, and Pop15=α·Any+(1−α)·Pop8. In all cases the 12 free populations act as auxiliaries. The outcomes have been ranked according to the value of φ and the first ones presented in Table 1 (upper block), where the nominal case leads. Note that none of the pre-JL values of ϕ, smaller than $90^{{}^{\circ}}$, initially point toward a mixing scenario unless we consider the possibility of a large effect of genetic drift. The linear fits are in Fig. 4 (top). $\Delta_{1}$ and $\Delta_{2}$ stand for 95% CI obtained from the linear fit (the first) and from the bootstrap on auxiliary populations (the second).

Using 850 300 SNPs the nominal admixture proportion is accurately reproduced, with φ close to $180^{{}^{\circ}}$ [last line (*) of the upper block of Table 1]. The effect of increasing the number of SNPs is also observed in the ranges of the regression plot in Fig. 4 (top), for the nominal case.

2. ACB.DG=α·GBR.SG+(1−α)·Yoruba.DG

This is a well-known historical example of an admixed group in the form of the Afro-Caribbean population (*ACB*), as an example to validate it as the product of admixture between a European source (represented by the *GBR* population as a proxy) and an African source (represented by the *Yoruba* population as a proxy). Twenty seven auxiliary populations have been chosen (see inset in Fig. 2).

The result for the slope α in Fig. 4 is the commonly accepted one. The estimates for both mixing angles in Table 1, $\varphi \gg \phi >90^{{}^{\circ}}$, confirm that the model corresponds to a real mixture, with a large reduction in genetic drift. Furthermore, φ close to $180^{{}^{\circ}}$ indicates that the sources are close to the exact ones.

If instead we choose the combination: $\mathrm{Yoruba}.DG=\alpha^{\prime }\cdot \mathrm{GBR}.SG+(1-\alpha^{\prime })\cdot \mathrm{ACB}.DG$, then the resulting linear fit (not shown) is as good as the model above, with slope: $\alpha^{\prime }=-\alpha /(1-\alpha )=-0.12$; and $\phi =72^{{}^{\circ}}>\varphi =4^{{}^{\circ}}$. Thus, a good regression plot with slope out of range can hide a potentially valid triplet in an alternative combination.

3. ACB.DG=α·Han.SG+(1−α)·Brahui.DG

This model is unrealistic because neither *Han* nor *Brahui* are close to the actual parentals, which is illustrated by the distribution of points in Fig. 4 where the linear regression is not even necessary. In any case, the confirmation can be found in Table 1: negative slope and φ<ϕ.

## 5 Conclusion

*Mixtum* offers assistance in the study of two-way admixture in population genetics within the framework of *f*-statistics. It allows interactive testing of different models using arbitrary sets of auxiliary populations, tracking the role of each one. The estimate of the admixture proportions is buttressed by the value of the angle that attests to the reconstruction quality of the ancestral alignment of the model, a novel indicator in the *f*-statistics formalism. Along with the visual environment, this is the main difference from the available *f*-statistics packages.

## Data Availability

The curated dataset of human genotypes from Allen Ancient DNA Resource (AADR), v62.0_1240k, is available at https://doi.org/10.7910/DVN/FFIDCW. The simulated dataset is available with the *Mixtum* software at https://doi.org/10.5281/zenodo.17789375 and https://github.com/jmcastelo/mixtum.
